# Pre-clinical studies of EC2629, a highly potent folate- receptor-targeted DNA crosslinking agent

**DOI:** 10.1038/s41598-020-69682-9

**Published:** 2020-07-29

**Authors:** Joseph A. Reddy, Melissa Nelson, Christina Dircksen, Marilynn Vetzel, Theresa Johnson, Vicky Cross, Elaine Westrick, LongWu Qi, Spencer Hahn, Hari Krishna Santhapuram, Garth Parham, Kevin Wang, Jeremy F. Vaughn, Albert Felten, Michael Pugh, June Lu, Patrick Klein, Iontcho R. Vlahov, Christopher P. Leamon

**Affiliations:** Endocyte, Inc., 3000 Kent Ave., Suite A1-100, West Lafayette, IN 47906 USA

**Keywords:** Drug discovery, Oncology

## Abstract

Folate receptor (FR)-targeted small molecule drug conjugates (SMDCs) have shown promising results in early stage clinical trials with microtubule destabilizing agents, such as vintafolide and EC1456. In our effort to develop FR-targeted SMDCs with varying mechanisms of action, we synthesized EC2629, a folate conjugate of a DNA crosslinking agent based on a novel DNA-alkylating moiety. This agent was found to be extremely potent with an in vitro IC50 ~ 100× lower than folate SMDCs constructed with various microtubule inhibitors. EC2629 treatment of nude mice bearing FR-positive KB human xenografts led to cures in 100% of the test animals with very low dose levels (300 nmol/kg) following a convenient once a week schedule. The observed activity was not accompanied by any noticeable weight loss (up to 20 weeks post end of dosing). Complete responses were also observed against FR-positive paclitaxel (KB-PR) and cisplatin (KB-CR) resistant models. When evaluated against FR-positive patient derived xenograft (PDX) models of ovarian (ST070), endometrial (ST040) and triple negative breast cancers (ST502, ST738), EC2629 showed significantly greater anti-tumor activity compared to their corresponding standard of care treatments. Taken together, these studies thus demonstrated that EC2629, with its distinct DNA reacting mechanism, may be useful in treating FR-positive tumors, including those that are classified as drug resistant.

## Introduction

The folate receptor (FR) is a cell surface receptor that is overexpressed by many primary and metastatic cancers, including ovarian, lung and breast cancers^1–4^. Covalent conjugation of the vitamin folic acid to various therapeutic- and imaging-based agents has enabled their specific delivery to tumors that express the FR protein^5–7^. Therefore, we have been developing folate-targeted small molecule drug conjugates (SMDC’s) to potentially enhance the safety and efficacy of anti-cancer agents^8–16^.

Pyrrolobenzodiazepine (PBD) dimers are a relatively new class of anticancer agents which bind to the minor groove of DNA, where they form covalent aminal crosslinks between the guanine residues with the two imine groups of the PBD^17^. The resulting PBD-DNA crosslinks halt progression of replication forks and arrest tumor cells at the G2-M boundary, leading to cellular apoptosis. The unusually high potency of PBD dimers is due to their cell cycle–independent activity and to their minimal distortion of DNA, increasing the chances of evasion of DNA damage repair mechanisms^18^. PBD compounds have been tested against a variety of tumors including ovarian cancer, SCLC (small cell lung cancer), and AML (acute myeloid leukemia). The investigational molecule, SJG136, was the first form of PBD dimer tested in clinical studies^19^, and this untargeted agent was found to be associated with dose-limiting hepatotoxicity and vascular leak syndrome. In an attempt to reduce toxicity, antibody drug conjugates (ADCs) of PBD’s have been designed, thus combining the potent antitumor activity of the PBD dimer with the targeting ability of the antibody. Two such agents under clinical evaluation are vadastuximab talirine and rovalpituzumab tesirine (Rova-T) that target CD33a in AML and DLL3 in SCLC, respectively^20,21^.

As presented herein, we have developed an SMDC of a modified pro-PBD dimer with more desirable physicochemical and pharmacokinetics properties than ADC’s, with the expectation that it would selectively kill FR-expressing tumor cells and possibly limit systemic toxicities. Hence, we report on our detailed in vivo investigation of a folate pro-PBD dimer conjugate (EC2629), with particular emphasis on effectively targeting and eradicating FR-expressing conventional, PDX and drug resistant tumors.

## Results

### Chemical structure of EC2629

**Figure 1:**
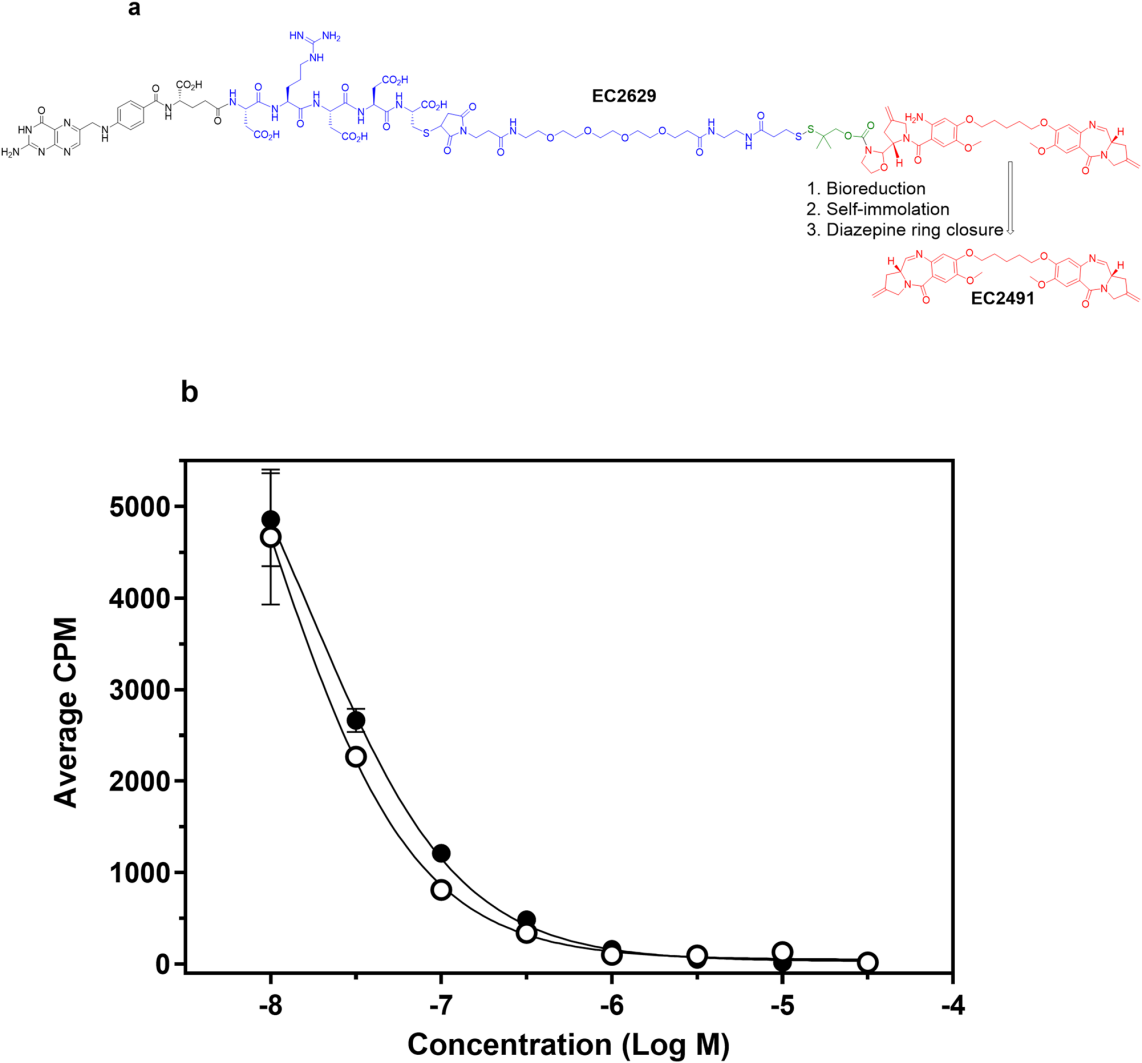
Chemical structure and relative folate receptor binding affinity of EC2629. (**a**) Module 1 (in black) is the tumor-targeting ligand, folic acid. Module 2 (in blue) is a hydrophilic peptidic-PEG spacer. Module 3 (in green) is a bio-cleavable, self-immolative protected disulfide-based linker system. Module 4 (in red) is a pro-PBD which when released after disulfide reduction forms the PBD dimer agent, EC2491. (**b**) KB cells were incubated for 1 h at 37 °C with 100 nmol/L [^3^H]-FA in the presence of increasing competitor concentrations. open circle, FA; filled circle, EC2629 and cell associated ^3^H measured. *Points*, average CPM of three wells; *bars,* standard deviation (s.d.).

Similar to other folate-based SMDCs, EC2629^22^ (Fig. 1a) was constructed using a modular design^23^. It contains the tumor-targeting ligand, folate (in black), a hydrophilic peptide-polyethylene glycol (PEG) spacer consisting of L-Asp-L-Glu-L-Cys-PEG4 (in blue), a self-immolative penicillamine protected disulfide (in green) and the pro-drug form of the potent-PBD dimer (in red). Once the prodrug (pro-PBD) conjugate enters a targeted cell, cleavage of the linker system triggers the generation of a reactive intermediate which goes through an intramolecular ring-closing reaction to form the second diazepine ring of the cytotoxic PBD dimer, EC2491^24^.

### High affinity FR-mediated binding of EC2629

The affinity of EC2629 for the FR was evaluated using a relative affinity assay that measures the ability of EC2629 to compete with folic acid for binding to cell surface FRs and further internalization. When folic acid’s affinity for human FR’s on KB cells was set to unity, our results show that EC2629 has a relative affinity of 0.53 (Fig. 1b). This demonstrated that conjugation of the folate targeting ligand to a hydrophilic spacer and a pro-PBD dimer minimally lowered the vitamin’s affinity for the FR.

### EC2629 crosslinks DNA only following release of free drug

**Figure 2:**
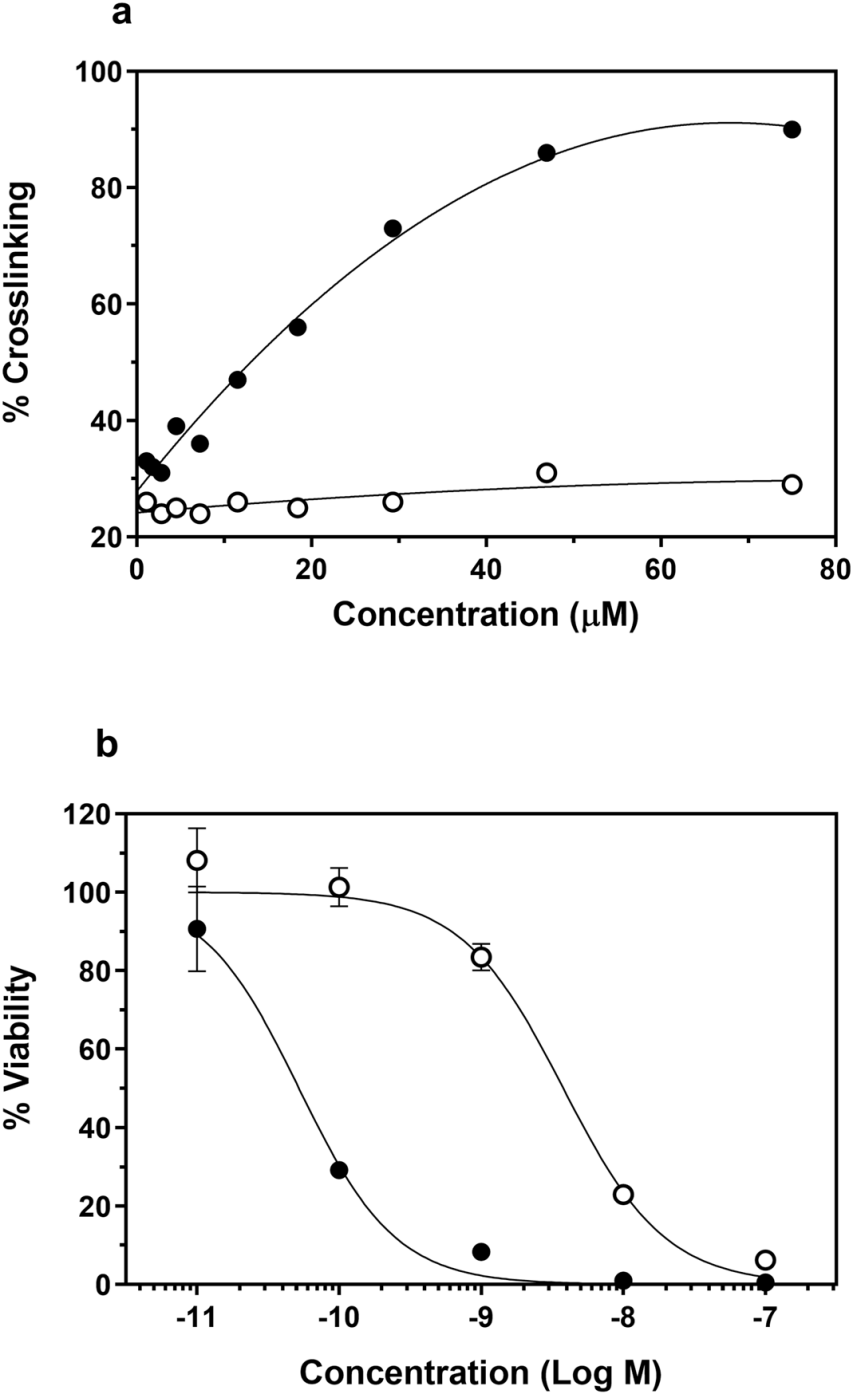
DNA interstrand crosslinking assay *and *in vitro activity of EC2629. (**a**) Calf thymus DNA mixed with varying concentrations of EC2629 with (filled circle) or without (open circle) 0.1 mM DTT were incubated at 37 °C for 2 h. Ethidium bromide was then mixed with above solution, incubated at room temperature for 2 h and fluorescence (Ex: 535 nm, Em: 605 nm) measured. The DNA solution was then heat denatured, cooled and fluorescence re-measured. The ratio of the fluorescence after heating to the fluorescence before heating yields the % DNA cross-linking. (**b**) KB cells were pulsed for 2 h with increasing concentrations of EC2629 in the absence (filled circle) or presence of 100 µM folic acid (open circle) as a competitor. *Points*, average of three wells; *bars,* s.d.

The ability of EC2629 to crosslink DNA was investigated using a DNA interstrand crosslinking assay. To measure crosslinking, calf-thymus DNA was thermally denatured and cooled in the presence of ethidium bromide. Under these conditions, strands do not reanneal, except when they have been crosslinked, resulting in ethidium fluorescence enhancement. EC2629 in the presence of DTT (reduction to cleave the disulfide bond resulting in the formation of the active PBD dimer, EC2491) displayed a concentration-dependent increase in fluorescence, while intact EC2629 showed background fluorescence at all concentrations (Fig. 2a). This demonstrates that EC2629 acts as an inactive pro-drug with undetectable DNA binding ability until it is reduced to release the pro-PBD dimer with subsequent conversion to the active DNA crosslinking molecular form. Although this data shows that 15 µM of EC2629 crosslinks 50% of the DNA, cytotoxic intracellular concentrations of PBD dimers are expected to be much lower. For instance, by quantifying the number of DNA interstrand cross-linked adducts in an in vivo xenograft model dosed with an ADC conjugate of a PBD dimer, the authors estimated that 1.2 × 10^4^ PBD-dimers per cell were very efficacious^25^. Since most FR positive tumors can reach 4 × 10^6^ receptors/cell, the amount of deliverable PBD-dimer should be well above the required threshold.

### EC2629 displays targeted picomolar potency against FR-expressing cells in vitro

Dose response activity and specificity of EC2629 were evaluated in vitro. As shown in Fig. 2b, FR-positive KB cells were found to be highly sensitive to EC2629 with an IC_50_ of 52 +/− 1.1 pM. The folate receptor targeted specificity of cell kill was demonstrated by the ~ 100-fold decreased potency when EC2629 was competed with an excess of folic acid. EC2629 has been selected from twenty new folate targeted PBD’s synthesized at Endocyte and IC50’s of all these agents were in the range of 30 pM–2 nM, when tested in varied FR expressing cell lines. We have also compared the activities of previously published folate conjugates of microtubule inhibitors with EC2629 in Supplementary Table S1 and found EC2629 to be ~ 100× more potent than those folate SMDC’s.

### EC2629 displays potent anti-tumor activity against KB tumor xenografts in mice and rats

**Figure 3:**
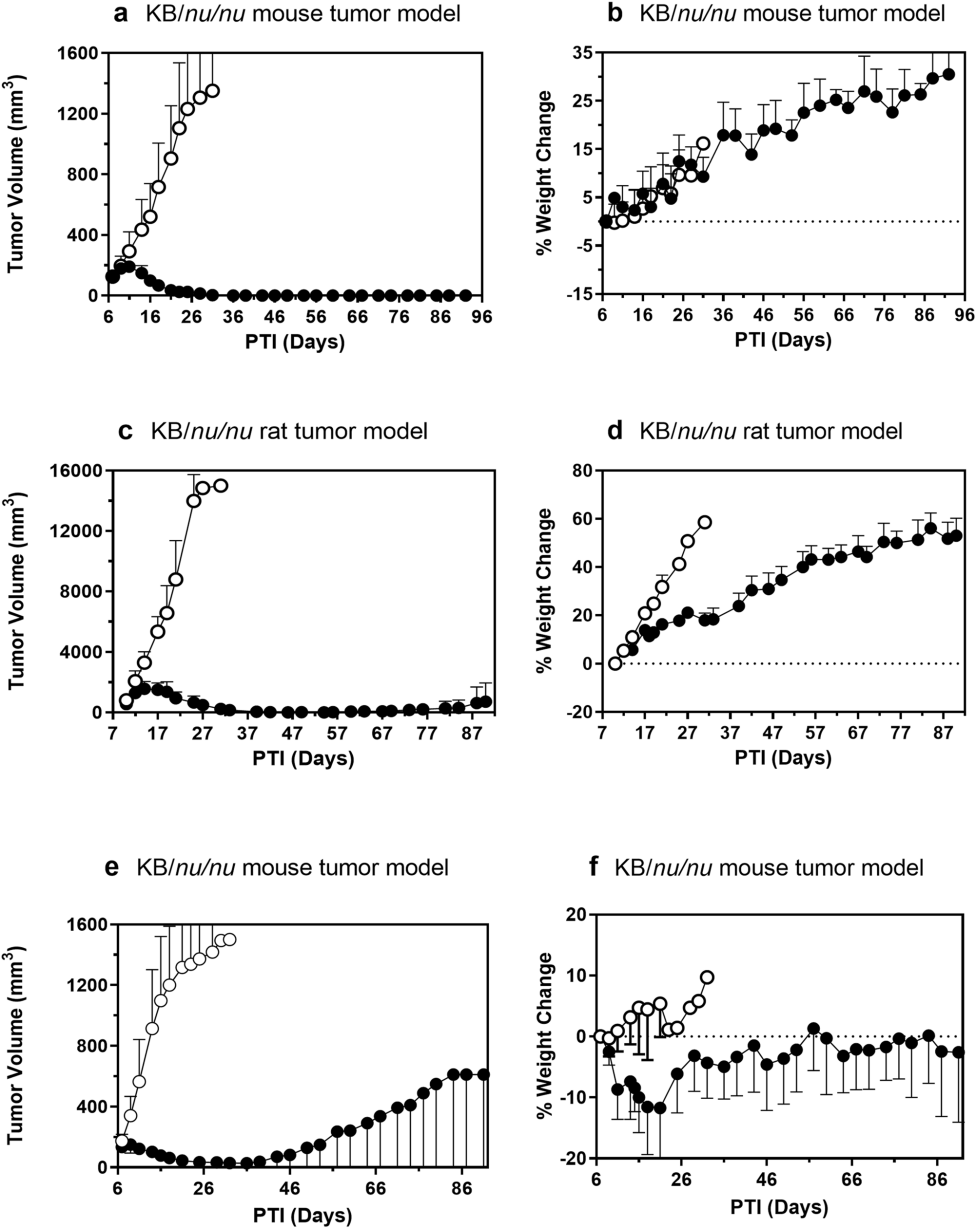
Antitumor (**a**, **c**, **e**) and weight change (**b**, **d**, **f**) effects of EC2629 (**a**, **b**, **c**, **d**) and SJG-136 (**e**, **f**) on FR expressing KB tumor *nu/nu* mice (**a**, **b**, **e**, **f**) and rat (**c**, **d**) models. KB tumor cells were inoculated subcutaneously into *nu/nu* mice (1 × 10^6^ cells) and rats (5 × 10^6^ cells) and therapy started on randomized animals with tumors in the 111–168 mm^3^ (mice; n = 5) and 411–704 mm^3^ (rats; n = 3) range. open circle, untreated controls; filled circle, EC2629, 0.3 µmol/kg (**a**, **b**) or 0.15 µmol/kg (**c**, **d**) or SJG-136, 0.3 mg/kg (**e**,**f**) in 0.05% Tween-80) SIW × 2 weeks. Each curve shows the average volume of 3–5 tumors/animals; bars, s.d.

We have used the FR-positive parental KB tumor model as a consistent standard against which we test and fairly compare folate SMDCs with varying payloads, linkers and spacers across many years of discovery efforts. Hence, the activity of EC2629 against this model was assessed by intravenously treating mice with 0.3 µmol/kg and rats with 0.15 µmol/kg following a once a week (SIW), 2-week schedule. Mice and rats were divided into two groups each and treatments started when the tumors had reached the 111–168 mm^3^ (mice) or 411–704 mm^3^ range (rats). Untreated control mice reached a tumor size of 1,500 mm^3^ by 23–30 days PTI (post tumor implant), whereas treatment with EC2629 led to 100% cures (Fig. 3a and Supplementary S1a). Control tumors in rats reached the target tumor size of 15,000 mm^3^ by 25–28 days PTI, while EC2629-treated rats yielded 2 CRs and 1 PR (Fig. 3c and S1c). Further, EC2629-treated animals did not lose any significant weight (Figs. 3b, d, S1b and S1d) throughout the dosing period and beyond, which is similar to that seen with our previously reported folate-targeted cytotoxic agents^8–10^. When mice were tested with SJG-136 (a clinically tested non-targeted PBD-dimer) at a previously reported dose of 0.3 mg/kg, an average weight loss of ~ 10% was observed with one death at 2 weeks post start of treatment. In the remaining 4 mice, there were 2 cures and 2 PR’s (Figs. 3e, f, S1e and S1f). Thus, low dose levels of our targeted pro-PBD conjugate, given at an infrequent once a week schedule, produced cures in mice and almost cured large tumor bearing rats. The low doses needed to achieve cures precluded any noticeable weight loss or gross toxicity. These results show that EC2629 was equally active on small as well as large tumors and also in both the mouse and rat species. In comparison, untargeted PBD-dimer SJG-136, caused severe toxicity and death while yielding greatly compromised anti-tumor activity. Combined with the demonstrated ability of EC2629 to crosslink DNA and its targeted cytotoxic activity on FR expressing cells, it is our belief that similar PBD-DNA interstrand crosslinked adducts are responsible for the in vivo antitumor activity of EC2629 as has been reported for various PBD’s and ADC-PBD’s^26^.

### EC2629 is active against taxane and platin-resistant tumor xenograft models

**Figure 4:**
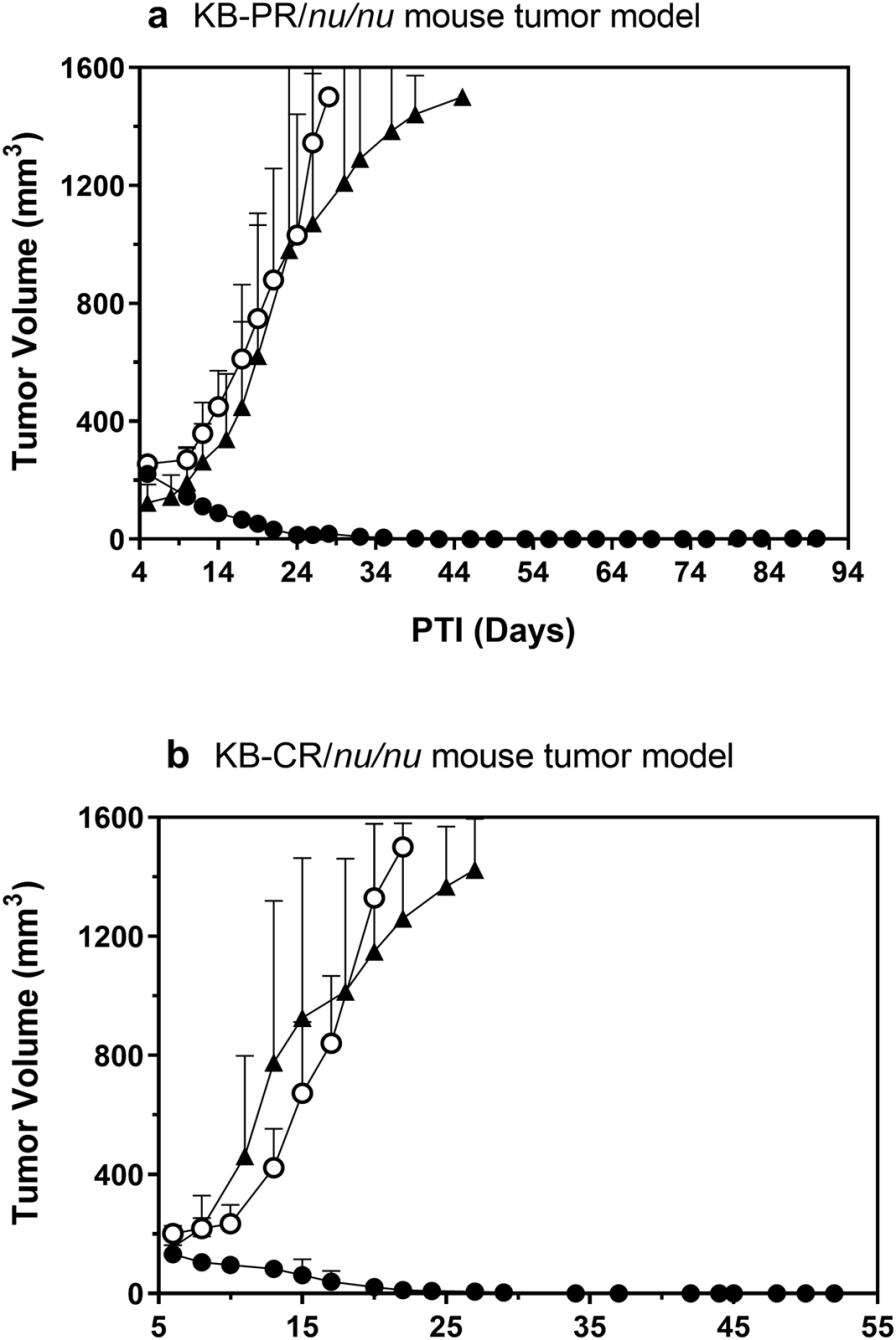
Antitumor effects of EC2629 on FR expressing KB-PR (**a**) and KB-CR (**b**) tumors in *nu/nu* mice. KB-PR (**a**) or KB-CR (**b**) tumor cells (1 × 10^6^) were inoculated subcutaneously into *nu/nu* mice and therapy started on randomized animals with tumors in the 205–243 mm^3^ (**a**) and 98–168 mm^3^ (**b**) range. open circle, untreated controls; filled triangles (**a**), paclitaxel, 20 mg/kg, TIW × 2 weeks (**b**) cisplatin, 3 mg/kg, BIW × 2 weeks; filled circle, EC2629, 0.5 µmol/kg, SIW × 2 weeks. Each curve shows the average volume of 4–5 tumors; bars, s.d.

A majority of ovarian cancer patients receive a first-line combination regimen that comprises of a taxane and a platinum drug. To be able to pre-clinically guide folate SMDC’s activity in patients that have relapsed and developed drug-resistant disease^27,28^, we had previously created FR expressing paclitaxel (KB-PR) and cisplatin-resistant (KB-CR) cells^16^. These KB-PR and KB-CR cells were inoculated subcutaneously into mice and treatment started when tumors were in a range of 205–243 mm^3^ and 98–168 mm^3^, respectively. As shown in Fig. 4a, b, both paclitaxel and cisplatin had very little effect against the KB-PR and KB-CR tumors, respectively. Two doses of EC2629 at 0.5 µmol/kg following a once a week schedule was found to be highly active yielding 100% cures in both the paclitaxel- and cisplatin-resistant models (Fig. 4a, b, Supplementary S2a and S2b). Thus, EC2629 was again curative in both the low P-gp (P-glycoprotein) expressing cisplatin refractory model, and the higher P-gp expressing paclitaxel refractory tumor model^16^. This outcome indicated that the activity of EC2629 was predominantly independent of the levels of p-glycoprotein expression.

### EC2629 is active against patient-derived xenograft (PDX) models

**Figure 5:**
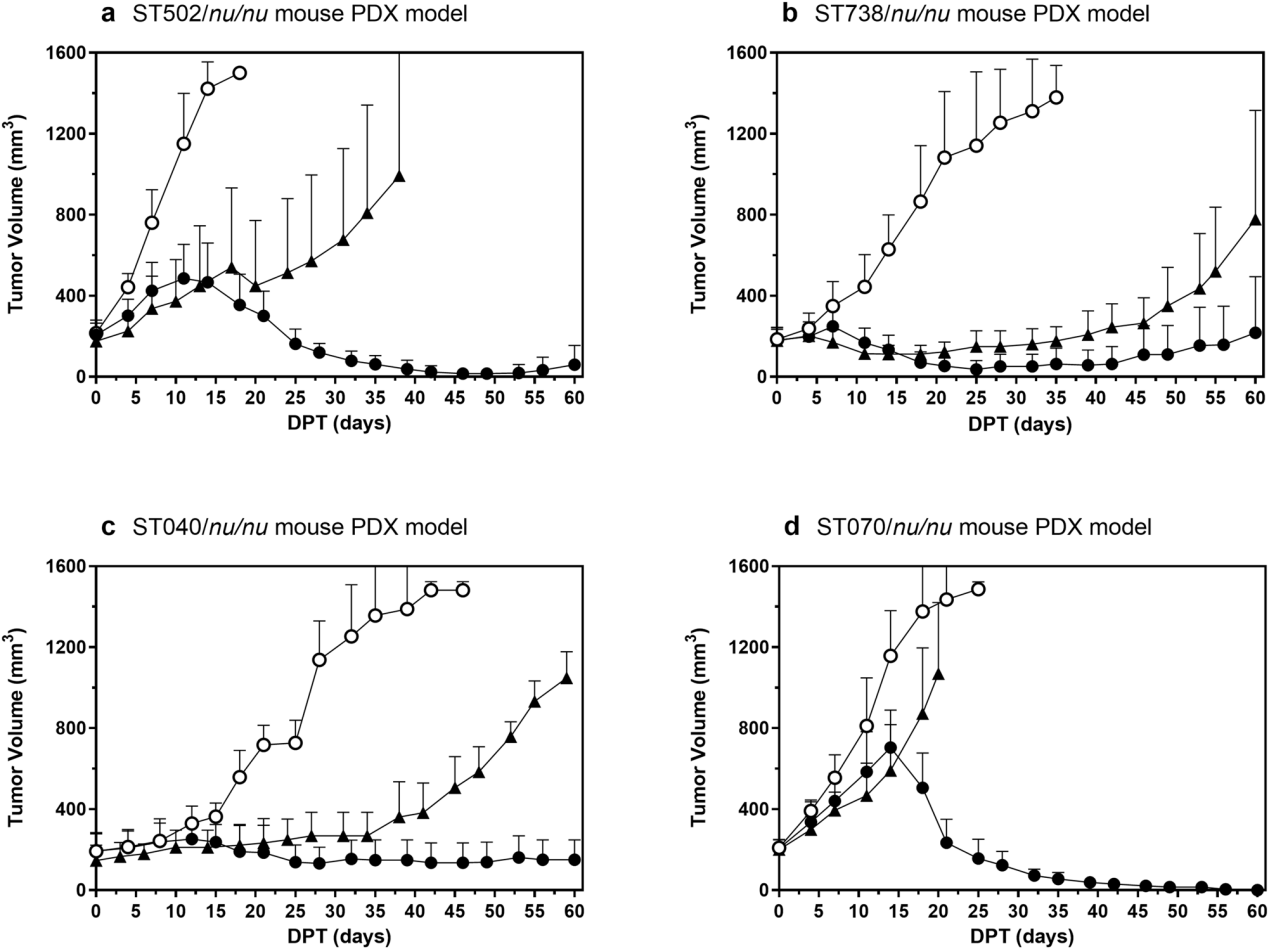
Antitumor effects of EC2629 on patient derived triple negative breast ST502 (**a**), ST738 (**b**), endometrial ST040 (**c**) and ovarian ST070 (**d**) xenograft models in *nu/nu* mice. Tumor fragments harvested from host animals were inoculated subcutaneously into *nu/nu* mice and therapy started on randomized animals with tumors in the 112–333 mm^3^ (**a**), 112–300 mm^3^ (**b**), 78–333 mm^3^ (**c**) and 131–266 mm^3^ (**d**) range. open circle, untreated controls; filled triangle, eribulin mesylate, 1 mg/kg SIW × 2 weeks (**a**, **b**) or paclitaxel, 15 mg/kg, SIW × 2 weeks (**c**, **d**) filled circle, EC2629, 0.27 µmol/kg, BIW × 2 weeks (**a**, **b**, **c**, **d**). Each curve shows the average volume of 3–7 tumors; bars, s.d.

Clinically, folate targeted therapy would have its best chance against those cancers that overexpress the FR such as TNBC^29^, ovarian^30^ and endometrial cancers^31^. In addition to FR expression being significantly associated with poor prognosis, these cancers also have limited therapeutic options. Since PDX models have recently been a preferred pre-clinical choice for testing anti-cancer drug efficacy, EC2629 was tested on FR positive PDX models of these cancers. EC2629 was evaluated in comparison with standard of care agents against two basal like breast cancer PDX models [ST502,112–333 mm^3^ and ST738; 112 to 300 mm^3^], an endometrial serous papillary cancer model [ST040; 78 to 333 mm^3^] and one ovarian cancer model [ST070; 131–266 mm^3^]. In this study EC2629 was dosed at 0.3 µmol/kg (ST502) or 0.27 µmol/kg (ST738) twice a week (BIW) for 2 weeks, and eribulin mesylate was dosed at 1 mg/kg, SIW × 2 weeks against the breast cancer models (Fig. 5a, b, Supplementary S3a and S3b). In the ST502 model, eribulin mesylate did not display significant anti-tumor activity, producing one stable disease (SD) and one partial response (PR) among seven mice; whereas, EC2629 generated two PRs, two complete responses (CRs) and three cures among a different cohort of seven mice (Fig. 5a and S3a). In the ST738 model, eribulin mesylate therapy yielded five SDs and two PRs, whereas EC2629 yielded enhanced activity with two SDs, two PRs, one CR, and two cures (Fig. 5b and S3b).

In the ST040 endometrial and ST070 ovarian cancer models, EC2629 was evaluated at 0.27 µmol/kg, BIW × 2 weeks in comparison with paclitaxel at 15 mg/kg, SIW × 2 weeks. As shown in Fig. 5c and S3c, paclitaxel only showed modest tumor growth delay in the ST040 model with no SD’s in that cohort, whereas EC2629 displayed stronger anti-tumor effect with two SDs and three PRs among the 7 mice (Fig. 5c and S3c). We observed more striking results in the ST070 model where paclitaxel was essentially inactive, whereas EC2629 yielded a 100% cure rate (Fig. 5d and S3d).

In summary, when compared with standard of care agents for each cancer type, EC2629 was found to be highly active and outperformed eribulin mesylate in two triple negative breast cancer models as well as paclitaxel in endometrial and ovarian cancer models. Generally, EC2629’s anti-tumor activity in these PDX models was found to be independent of their sensitivity to standard-of-care chemotherapy regimens.

### EC2629 combines well with anti-CTLA-4 antibody against a syngeneic FR-positive mouse ovarian cancer model

**Figure 6:**
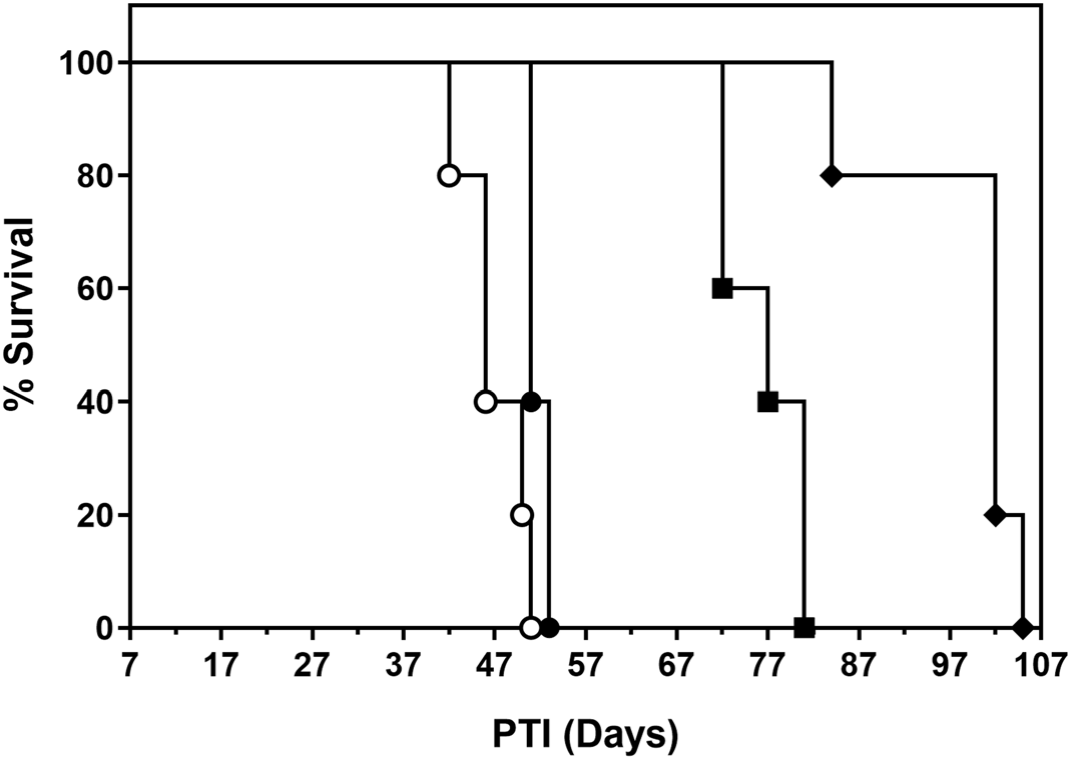
Antitumor efficacy of EC2629 +/− anti-CTLA-4 on FR expressing intraperitoneal ID8-Cl15 tumors in C57BL/6 mice. ID8-Cl15 tumor cells (1 × 10^6^) were inoculated intraperitoneally into C57BL/6 mice and therapy administered on randomized animals. open circle, untreated controls; filled circle, anti-CTLA-4 antibody 0.25 mg/dose, BIW, 5 doses; filled square, EC2629, 0.1 µmol/kg, BIW × 6 doses; filled diamond, EC2629+ anti-CTLA-4 antibody. N = 5 mice/group.

Current practice in the clinic is to utilize multidrug combinations instead of single-agent chemotherapy. Such regimens are intended to benefit from the non-overlapping tissue toxicity combined with their antitumor activity against differentially resistant cancer cell clones. Immune checkpoint inhibitors have greatly improved the therapeutic outcome of several cancer types^32^, while displaying a mild toxicity profile that appeared to be varied from that of PBD’s. It was therefore hypothesized that combining EC2629 with an immune checkpoint inhibitor may afford greater therapeutic efficacy compared to single-agent therapy. The anti-cytotoxic T lymphocyte antigen-4 (CTLA-4) blocking antibody ipilimumab was the first immune checkpoint inhibitor to be approved. CTLA-4 is a key negative regulator of T cell activation, and CTLA-4-blocking antibodies have been shown to enhance the antitumor activity of chemotherapy^33^. Hence, in the following study EC2629 was evaluated as a single agent and in combination with the anti-CTLA-4 antibody against the FR-expressing ID8-Cl15 intraperitoneal C57BL/6 mouse syngeneic model. Starting 7 days after tumor implant, EC2629 (0.1 µmol/kg, BIW for 3 weeks) was administered intravenously alone and in combination with anti-CTLA-4 antibody (250 µg, BIW for 2.5 weeks). As shown in Fig. 6, untreated control mice and those treated with anti-CTLA-4 alone had a median survival time of ~ 46 days and 51 days PTI, respectively. However, single agent EC2629 therapy displayed greater anti-tumor effect with ~ 67% increase in the median survival time (~ 77 days PTI). In addition, EC2629 combined very well with the anti-CTLA-4 antibody resulting in a median survival time of ~ 102 days PTI. Because immune checkpoint inhibitors work by removing the “brakes” on the immune system rather than by directly killing tumor cells, patients may also benefit from combination therapies that include highly potent targeted cytotoxic molecules, such as EC2629, which directly interfere with tumor cell growth.

### Pharmacokinetics in rat

Plasma EC2629 mean concentration–time profiles after dosing males and females with EC2629 were qualitatively similar, with concentrations declining with time in a manner characteristic of intravenous dosing. EC2629 plasma C_max_ were generally similar for females and males of each dose group. The C_max_ for plasma EC2629 increased with dose, and this increase was generally dose-proportional. EC2629 plasma AUC_last_ estimates were generally higher for males than females of each dose group. EC2629 AUC_last_ also incrementally increased with dose for males and females. Estimates of volume of distribution (V_z_) were generally higher for males than females of each dose group. V_z_ ranged from 0.109–0.191 L/kg (Table 1). There was no apparent dose-related effect on V_z_. Clearance (Cl) estimates were generally higher for females than males of each dose group. Cl ranged from 0.0907 to 0.174 L/h/kg. Clearance values decreased with increasing dose for females, but the dose-related effect on clearance for males was more variable.

**Table 1:** EC2629 plasma pharmacokinetic parameters following intravenous administration to rats.

Group	Dose (mg/kg)	Gender	t_1/2_ (h)	T_max_ (h)	C_max_ (ng/mL)	C_0_ (ng/mL)	AUC_last_ (h · ng/mL)	AUC_inf_ (h · ng/mL)	V_z_ (L/kg)	Cl (L/h/kg)
1	0.05	F	0.627	0.033	545	662	260	287	0.157	0.174
		M	1.02	0.033	595	669	305	384	0.191	0.130
2	0.10	F	0.525	0.033	1,330	1,570	596	633	0.120	0.158
		M	0.706	0.033	1,390	1,690	617	699	0.146	0.143
3	0.25	F	0.530	0.033	3,230	3,740	1,640	1,750	0.109	0.143
		M	0.770	0.033	3,580	4,310	1,670	1930	0.144	0.129
4	0.50	F	0.611	0.033	6,070	7,060	3,230	3,540	0.125	0.141
		M	1.40	0.033	7,080	8,450	5,450	5,510	0.183	0.0907

Mean EC2491 concentrations were quantifiable through 12 h (t_last_) post-dose for animals in the 0.05 mg/kg dose group (Group 1), and through 24 h for animals in groups 2–4. Time of maximal observed plasma concentration (t_max_) was 0.033 h (2 min), the first sample collection time point, in all cases except for females in the 0.10 mg/kg dose group. Within the scope of the limited data available, no gender-, day-, or dose-related relationship could be established for terminal half-life (t_1/2_) estimates for plasma EC2491. Half-lives ranged from 3.09 to 10.0 h. EC2491 plasma C_max_ AUC_last_ estimates were generally higher for females than males of each dose group. C_max_ for plasma EC2491 increased with dose for males and females.

### Protein binding and stability in biological matrices

As presented above, EC2629’s favorable biological performance against a variety of different tumor models provides support for potential clinical evaluation. Prior to the start of preclinical development studies, EC2629’s protein binding and stability across a diverse set of biological matrices was evaluated.

#### Plasma protein binding

In vitro protein binding assay was used to estimate the percent protein bound EC2629 and EC2491 in plasma from multiple species. Results from this study indicated that EC2629 at 500 nM was bound to plasma protein at 87.2–97.4% across all species. EC2629 plasma protein binding was highest in rat at 97.4%, followed by mouse and dog plasma at 94.9% and 94.5%, respectively. Human plasma protein binding was found to be the lowest at 87.2%. Thus, the effect of protein binding on the bioavailable portion of EC2629 in circulation should be quite similar across species. For comparison, the untargeted PBD dimer, EC2491, was found to be highly bound (95.0–98.0%) to plasma proteins across all species tested.

#### EC2629 and EC2491 plasma and whole blood stability

In vitro plasma and whole blood stability of EC2629 and EC2491 was evaluated for multiple species by monitoring the percent of each compound remaining over two hours at 37 °C. In plasma analysis, EC2629 at 500 nM concentration was found to be stable in rat, mouse, dog, and human plasma, with minimal observed degradation during the 2 h experiment. EC2491 was stable in rat, mouse, and dog plasma for greater than 24 h. In whole blood analysis, EC2629 was most stable in mouse and human blood, with no observed degradation over the experimental time interval. EC2629 was also relatively stable in rat and dog whole blood, with 75.1% and 75.8% remaining after two hours, respectively. Some degradation of EC2491 was observed in whole blood from each species. EC2491 was most stable in rat blood with 95.6% remaining after two hours, followed by 87.3% and 80.6% intact compound remaining in mouse and human whole blood, respectively. EC2491 was least stable in dog whole blood, with 63.1% remaining at the conclusion of the experiment. Results from the plasma and whole blood stability evaluations indicate that 500 nM EC2629 and EC2491 are stable across all species over two hours, which is a pharmacologically relevant time interval.

#### In vitro metabolic stability of EC2491

**Figure 7:**
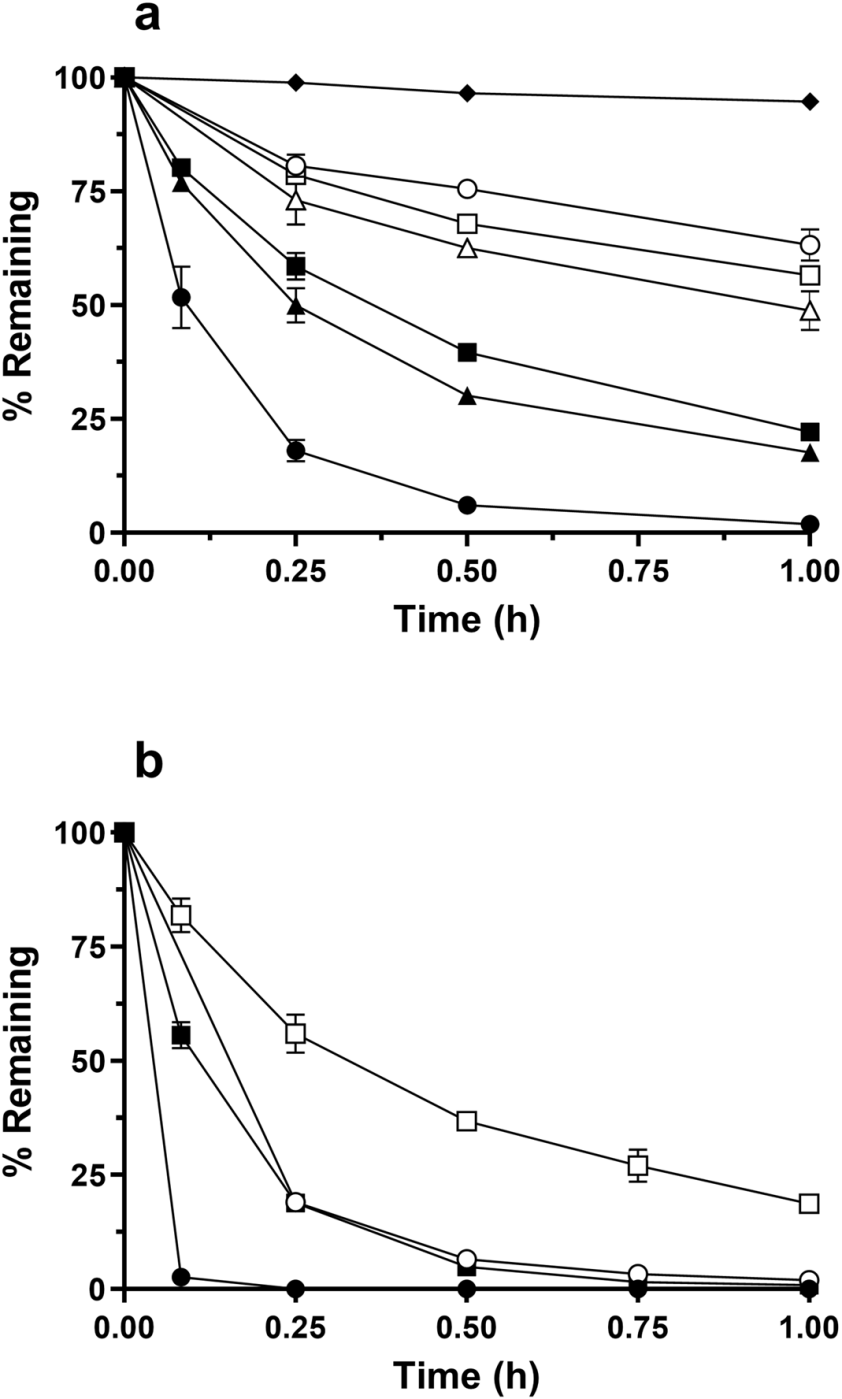
Stability of EC2491 in liver microsomes. Percent intact EC2491 after incubating 1 µM in (**a**) buffer (filled diamond), liver microsomes (rat, open circle; dog, open square; and human, open triangle) and liver microsomes + NADPH (rat, filled circle; dog, filled square; and human, filled triangle) and (**b**) liver microsomes (+ NADPH) from dexamethasone treated rat (filled circle), corn-oil treated rat (open circle), rifampin treated dog (filled square) or corn-oil treated dog (open square). *Points*, average of 2 samples; *bars,* s.d.

The in vitro metabolic stability of EC2491 was evaluated using rat, dog, and human liver microsomes in the presence and absence of NADPH. Stability of EC2491 was also evaluated in phosphate buffer, pH 7.4, under the incubation conditions (see Fig. 7a). EC2491 (1 µM) is stable in 0.1 M phosphate buffer, pH 7.4 up to 1 h at 37 °C. When EC2491 (1 µM) is incubated with male rat, dog, and human microsomes in the absence of NADPH, there is some depletion of the compound such that approximately 63%, 56%, and 49% of the compound remains after 1 h in rat, dog, and human microsomes, respectively. This result is indicative of some non-CYP mediated metabolism of EC2491 in the microsomes. In the presence of NADPH male rat liver microsomes metabolize EC2491 rapidly such that only 2% of the compound remains in the incubation mixture at the end of 1 h. Male dog and human liver microsomes metabolize EC2491 somewhat slowly (22% and 18% remaining at the end of 1 h respectively). This is indicative of CYP-mediated metabolism of EC2491.

EC2491 was also incubated with dexamethasone-treated male rat and rifampin-treated dog liver microsomes and their corresponding controls (corn-oil treated rat and dog liver microsomes) to evaluate the effect of CYP3A induction on metabolism of EC2491. Figure 7b depicts the stability of 1 µM EC2491 in these CYP3A induced microsomes in the presence of NADPH. Both, dexamethasone-treated rat, and rifampin-treated dog liver microsomes showed more rapid metabolism of EC2491 than their corresponding corn-oil treated controls, indicating that CYP3A induction could result in faster metabolism of EC2491 in rats and dogs, and potentially humans.

## Discussion

Aiming to reduce the reported off-target toxicities of clinically tested PBD agents such as SJG-136, vadastuximab talirine and Rova-T, we have masked one of the highly reactive imines of PBD in EC2629^22^. This should effectively block the imine moiety and prevent the intact molecule from alkylating and crosslinking DNA. Upon entering a targeted cell, endosomal reductive cleavage of the disulfide bond, fragmentation of the linker system and further spontaneous condensation should result in formation of the potent PBD molecule. We have also added a geminal-dimethyl functionality alpha to the disulfide bond on the linker. This structural feature was designed to further stabilize this SMDC during circulation by providing steric hindrance around the disulfide bond. In addition, the PK of an SMDC should ensure that the inactive prodrug is rapidly excreted from the body avoiding prolonged exposures and clearance that were associated with the PBD-ADC’s (half-life of Rova-T in humans was 10–14 days^21^.

Although clinical trials with folate targeted SMDC’s such as Vintafolide and EC1456 have been discontinued, a Phase 2 study of Vintafolide in advanced ovarian cancer subjects showed a tolerable safety profile. In addition, subjects in whom all target lesions were positive for FR expression (100% FR-positive) achieved a greater overall survival than those subjects who were less than 100% FR-positive^34^. This clinical observation leads us to believe that a Folate SMDC with a more potent warhead, such as a PBD, may potentially increase the therapeutic benefit in patients with fewer than 100% FR positive lesions or in patients with generally lower tumor FR expression levels.

In conclusion, EC2629 displays favorable biological properties including i) high affinity for the FR target, ii) sufficient stability in biological matrices, iii) highly potent and FR-specific cytotoxic activity, and iv) superior antitumor effect across numerous FR-expressing conventional, resistant and PDX tumor models following infrequent low dose schedules. Therefore, we present EC2629 as a promising first-in-class SMDC comprising a potent DNA crosslinking pharmacophore for the treatment of FR-positive tumors.

## Methods

### Materials

Folate-free RPMI media (FFRPMI) and PBS were obtained from Gibco, Grand Island, NY. ^3^H-thymidine was purchased from Moravek Biochemicals, Brea, CA. Anti-mouse CTLA-4 antibody (Clone UC10-4F10-11) was obtained from BioCell (West Lebanon, NH) All other common reagents were purchased from Sigma or other major suppliers. EC2629 was synthesized according to a published procedure^22^ and is covered by a patent application.

### Relative affinity assay

The relative affinity of EC2629 was determined according to a previously published procedure^35^. Briefly, 1 × 10^5^ FR-positive KB cells were seeded into each well of 24-well Falcon plates and allowed to form adherent monolayers overnight in FFRPMI/HIFCS. Spent incubation medium was replaced with FFRPMI/HIFCS containing 100 nM of ^3^H-FA in the absence and presence of increasing concentrations of unlabeled FA or EC2629. Cells were incubated for 1 h in triplicate wells at 37 °C and then rinsed 3 times with 0.5 mL of PBS. Five hundred microliters of 1% sodium dodecylsulfate in PBS were added to each well; after 5 min, cell lysates were collected, transferred to individual vials containing 5 mL of scintillation cocktail, and then counted for radioactivity. Cells exposed to only the ^3^H-FA were designated as negative controls, whereas cells exposed to the ^3^H-FA plus 1 mM unlabeled folic acid served as positive controls; DPMs measured in the latter samples (representing non-specific binding of label) were subtracted from the DPM values from all samples. Since these experiments were performed at 37 °C, the ^3^H values are a combination of receptor binding and cellular internalization of ^3^H-FA. This competitive relative affinity assay measures the ability of the test agent (EC2629) to compete with ^3^H-FA for binding to cell surface FRs and compares with folic acid’s ability to compete with itself (^3^H-FA).

### DNA interstrand crosslinking assay

Cross-linking reaction mixtures containing approximately 30 µg calf thymus DNA with varying concentrations (100 µM–100 nM) of EC2629 with or without 1 mM dithiothreitol (DTT) were first incubated at 37 °C for 2 h. Ethidium bromide (1 µg/ml) was then mixed with above solution, incubated at room temperature for 2 h and fluorescence measured on a Fluoroskan II fluorescent microplate reader at an excitation wavelength of 535 nm and emission wavelength at 605 nm. The DNA solution was then heat denatured at 104 °C for 5 min, rapidly cooled on ice for 5 min and fluorescence re-measured. The ratio of the fluorescence after heating to the fluorescence before heating gave the extent of covalent cross-linking.

### Dose-dependent FR-specific activity of EC2629

Parental KB cells (a human cell line from ATCC containing markers of HeLa cervical cancer origin) were seeded in individual 12-well Falcon plates and allowed to form nearly confluent monolayers overnight in folate-deficient RPMI medium supplemented with 10% fetal bovine serum. Following a detailed published procedure^15^, a 2 h pulse, 70 h chase assay format was used to evaluate the cytotoxic effects of increasing concentrations of EC2629. Viability was assessed by measuring ^3^H-thymidine incorporation into trichloroacetic acid precipitable material. Final results were expressed as the percentage of ^3^H-thymidine incorporation relative to untreated controls.

### In vivo antitumor experiments

Four- to eight-week-old female *nu/nu* mice and rats (Harlan Sprague Dawley, Inc.), were maintained on a standard 12-h light–dark cycle and fed ad libitum with a low-folate chow (Harlan Teklad diet #TD.01013, Madison, WI) for the duration of dosing and 1 week post dosing schedule^16^. Mice and rats were then switched to Teklad Global 18% Rodent diet (Harlan Teklad diet #2018S) during the monitoring phase of the study. Parental KB, KB-CR2000 or KB-PR10 cells (1 × 10^6^/100 µL per *nu/nu* mouse and 5 × 10^6^/200 µL per *nu/nu* rat) were injected into the subcutis of the dorsal medial area. Mice were divided into groups of 5 and rats in groups of 3–4, and freshly prepared test articles were injected through the lateral tail vein under sterile conditions in a volume of 200 μL/20 g of PBS. In mice, intravenous treatments typically initiated on day 7 PTI when KB tumors were approximately 111–168 mm^3^ in volume, on day 5 PTI when the KB-PR tumors were about 205–243 mm^3^, and on day 6 PTI when the KB-CR tumors were around 98 to 168 mm^3^. Treatments in rats were started on day 10 PTI when KB tumors were approximately 412–704 mm^3^ in volume.

Patient derived xenograft (PDX) studies were performed at South Texas Accelerated Research Therapeutics (START, San Antonio, TX). Patient tumor tissues were obtained with patients’ informed consent and PDX models were generated after multiple generations in mice. The four PDX models chosen for this study were shown to be strongly positive for FRα staining by a FRα IHC assay (Biocare Medical) at START. Tumor fragments were harvested from host animals and implanted into 6–12 weeks old female athymic nude mice (Crl:NU(NCr)-Foxn1nu). Intravenous treatments were initiated when ST502 (triple negative breast) tumors were approximately 112–333 mm^3^, ST738 (triple negative breast) tumors were 112–300 mm^3^, ST040 (endometrial) tumors were 78–333 mm^3^, and ST070 (ovarian) tumors were 131–266 mm^3^ in volume.

Animals in the control groups received no treatment. Growth of each subcutaneous tumor was followed by measuring the tumor 3 times per week during treatment and twice per week thereafter, until a maximum volume of 1,500 mm^3^ for mice and 15,000 mm^3^ for rats were reached. Tumors were measured in two perpendicular directions using Vernier calipers, and their volumes were calculated as V = 0.5 × L × W^2^, where L = measurement of longest axis in mm and W = measurement of axis perpendicular to L in mm^16^. As a general measure of gross toxicity, changes in body weights were determined on the same schedule as tumor volume measurements. Survival of animals was monitored daily. Animals that were moribund (or unable to reach food or water) were euthanized by CO_2_ asphyxiation. Individual tumor response endpoints were reported in terms of tumor volume change. A partial response (PR) was defined as volume regression > 50% but with measurable tumor (> 2 mm^3^) remaining at all times. Complete response (CR) was defined as a disappearance of measurable tumor mass (< 2 mm^3^) at some point within 90 days after tumor implantation. Cures were defined as CRs without tumor regrowth within the 90-day study time frame^8^.

ID8-Cl15, a mouse epithelial ovarian cancer cell line was transfected to express high levels of murine FR. Similar to ovarian cancer patients, syngeneic C57BL/6 mice with intraperitoneally implanted ID8-Cl15 cells developed severe ascites after a month. Seven days post tumor implant, EC2629 was administered intravenously and anti-CTLA-4 antibody was dosed intraperitoneally. The tumor bearing mice were euthanized when their ascites became severe based on the degree of abdominal distention, signs of distress and weight gain. All animal housing, care, and procedures were followed according to Purdue Animal Care and Use Committee (PACUC)-approved animal care and use protocols.

### Pharmacokinetics in rat

The plasma pharmacokinetics of EC2629 and metabolite EC2491 were evaluated following intravenous administration of EC2629 to male and female rats. The toxicokinetic phase of the study consisted of 5 groups with three animals/gender assigned to the control group, and nine animals/gender assigned to each of the treatment groups. EC2629 in 0.9% sodium chloride (saline) was administered at the following dosages: Control—0 mg/kg/dose (Group 0); 0.05 mg/kg/dose (Group 1); 0.1 mg/kg/dose (Group 2); 0.25 mg/kg/dose (Group 3); and 0.5 mg/kg/dose (Group 5). Blood samples were collected from Group 0 at 10 min post-dose, and from Groups 1–4 at pre-dose, 2, 10, and 30 min and 1, 2, 8, 12, and 24 h post-dose. Plasma was prepared and analyzed for concentrations of EC2629 and metabolite EC2491. Mean plasma EC2629 and EC2491 concentration–time data for males and females, separately, of Groups 1–4 and were subjected to noncompartmental toxicokinetic evaluations and the results are summarized in Table S3.

### Plasma protein binding

Plasma ultrafiltration was used for determination of free and protein bound fractions of EC2629 and EC2491. Samples were extracted and analyzed using fit-for-purpose bioanalytical methods. Briefly, 500 nM EC2629 or EC2491 was spiked into plasma maintained at 37 °C prior to plasma ultrafiltration. Plasma spiked with 500 nM EC2629 or EC2491 was allowed to equilibrate at 37 °C for 30 min prior to processing. Next, 50 μL of plasma was transferred to a clean 1.2 mL plate stored at 2–8 °C for further processing. The remaining plasma sample volumes were transferred to 30 Kd MWCO PES filter to generate plasma ultrafiltrate by centrifugation at 10,000×*g* for 20 min. After which, 50 μL of each plasma ultrafiltrate was transferred to separate wells in a clean 1.2 mL plate. To all spiked plasma samples, 50 μL of blank plasma ultrafiltrate was added. 50 μL of blank plasma was added to all experimental ultrafiltrate samples. These blank matrix additions normalized the matrix effects for protein binding determination. Samples were then extracted using protein precipitation with a 3:1 ratio of acetonitrile after addition of internal standard. The supernatant transferred to a new plate, evaporated, and reconstituted prior to LC–MS/MS analysis.

### EC2629 and EC2491 plasma and whole blood stability

Whole blood and plasma stability were determined for EC2629 and EC2491 at a concentration of 500 nM. Samples were extracted and analyzed using fit-for-purpose bioanalytical methods. EC2629 or EC2491 was spiked into 1 mL whole blood or plasma maintained at 37 °C for 30 min prior to use . Fifty µl aliquots were then removed at defined timepoints and stored in a 1.2 mL extraction plate prior to analysis. Samples were then extracted using protein precipitation with a 3:1 ratio of acetonitrile after addition of internal standard. The supernatant was then transferred to a new plate, evaporated, and reconstituted prior to LC–MS/MS analysis.

### Liver microsome stability assay

Appropriate microsomes (XenoTech) were incubated with EC2491 in the presence or absence of NADPH regenerating system (Corning Gentest) at 37 °C. Incubations with NADPH contained 352.5 μL DI water, 100 μL 0.5 M potassium phosphate buffer, pH 7.4, 25 μL NADPH solution A, 2 μL solution B, and 12.5 μL of the appropriate microsomes. Incubations without NADPH contained 382.5 μL water, 100 μL 0.5 M potassium phosphate buffer, pH 7.4, and 12.5 μL of the appropriate microsomes. 50 μL aliquots were withdrawn at various time points and mixed with internal standard, EC3044 (Endocyte) in acetonitrile. After mixing and evaporation of the acetonitrile, the samples were reconstituted. The samples were mixed, centrifuged, and 100 μL of supernatant was transferred to a new plate prior to LC–MS/MS analysis.

## Supplementary information

Supplementary information.

## Data Availability

The datasets generated during the current study are available from the corresponding author on reasonable request.
