# Supplementary material for: Pre-clinical studies of EC2629, a highly potent folate- receptor-targeted DNA crosslinking agent
Source: Sci Rep. 2020 Jul 29;10:12772. doi: 10.1038/s41598-020-69682-9 (PMC7391724; doi:10.1038/s41598-020-69682-9)
Supplement: Supplementary file 1 — Supplementary information. [file 41598_2020_69682_MOESM1_ESM.pdf]

Pre-clinical studies of EC2629, a highly potent folate- receptor-targeted DNA crosslinking agent

Joseph A. Reddy[1], Melissa Nelson[1], Christina Dircksen[1], Marilynn Vetzel[1], Theresa Johnson[1], Vicky Cross[1], Elaine Westrick[1], LongWu Qi[1], Spencer Hahn[1], Hari Krishna Santhapuram[1], Garth Parham[1], Kevin Wang[1], Jeremy F. Vaughn[1], Albert Felten[1], Michael Pugh[1], June Lu[1], Patrick Klein[1], Iontcho R. Vlahov[1] and Christopher P. Leamon[1]

Supplementary Information:

Supplementary Table S1. Relative potencies of base drugs and associated folate-based SMDCs on KB cells

Base Drug			SMDC		
Name	*IC_{50} (pM)*	*Relative Potency*	*Name*	*IC_{50} (pM)*	*Relative Potency*
DAVLBH	35,000	1	EC145	9,000	1
EC0347	2500	14	EC1456	1500	6
EC2491	15	2333	EC2629	52	173

Supplementary Table S2: Activity and response for each of the graphs in figures 3, 4 and 5. SD, stable disease defined as volume regression < 50% and increase in volume of < 10% ; PR, partial response defined as volume regression >50% but with measurable tumor (>2 mm^3) remaining at all times; CR, complete response defined as a disappearance of measurable tumor mass (<2 mm^3) at some point within 90 days after tumor implantation; cures were defined as CRs without tumor regrowth within the 90-day study time frame.

Figure # : Tumor model	Treatment Regimen	n	SD	PR	CR	Cures
**Fig 3a: KB/ *nu/nu* mice**	**Control mice**	4	0	0	0	0
	**EC2629** 0.3 µmol/kg SIW x 2	5	0	0	0	5
**Fig 3c: KB/ *nu/nu* rats**	**Control rats**	3	0	0	0	0
	**EC2629** 0.15 µmol/kg SIW x 2	3	0	1	2	0
**Fig. 3e: KB/ *nu/nu* mice**	**Control mice**	5	0	0	0	0
	**SJG-136** 0.3 mg/kg SIW x 2	5	0	2	0	2
**Fig. 4a: KB-PR/ *nu/nu* mice**	**Control mice**	5	0	0	0	0
	**Paclitaxel** 20 mg/kg, TIW x 2	5	1	0	0	0
	**EC2629** 0.5 µmol/kg SIW x 2	5	0	0	0	5
**Fig. 4b: KB-CR/ *nu/nu* mice**	**Control mice**	4	0	0	0	0
	**Cisplatin** 3 mg/kg, BIW x 2	5	0	0	0	0
	**EC2629** 0.5 µmol/kg SIW x 2	5	0	0	0	5
**Fig. 5a: ST502/ *nu/nu* mice**	**Control mice**	7	0	0	0	0
	**Eribulin mesylate** 1 mg/kg SIW x 2	7	1	1	0	0
	**EC2629** 0.27 µmol/kg BIW x 2	7	0	2	2	3
**Fig. 5b: ST738/ *nu/nu* mice**	**Control mice**	7	0	0	0	0
	**Eribulin mesylate** 1 mg/kg SIW x 2	7	5	2	0	0
	**EC2629** 0.27 µmol/kg BIW x 2	7	2	2	1	2

**Fig. 5c: ST040/ *nu/nu* mice**	**Control mice**	7	0	0	0	0
	**Paclitaxel** 15 mg/kg SIW x 2	3	0	0	0	0
	**EC2629** 0.27 µmol/kg BIW x 2	7	2	3	0	0
**Fig. 5d: ST070/ *nu/nu* mice**	**Control mice**	7	0	0	0	0
	**Paclitaxel** 15 mg/kg SIW x 2	7	0	0	0	0
	**EC2629** 0.27 µmol/kg BIW x 2	7	0	0	0	5

Supplementary Figure S1

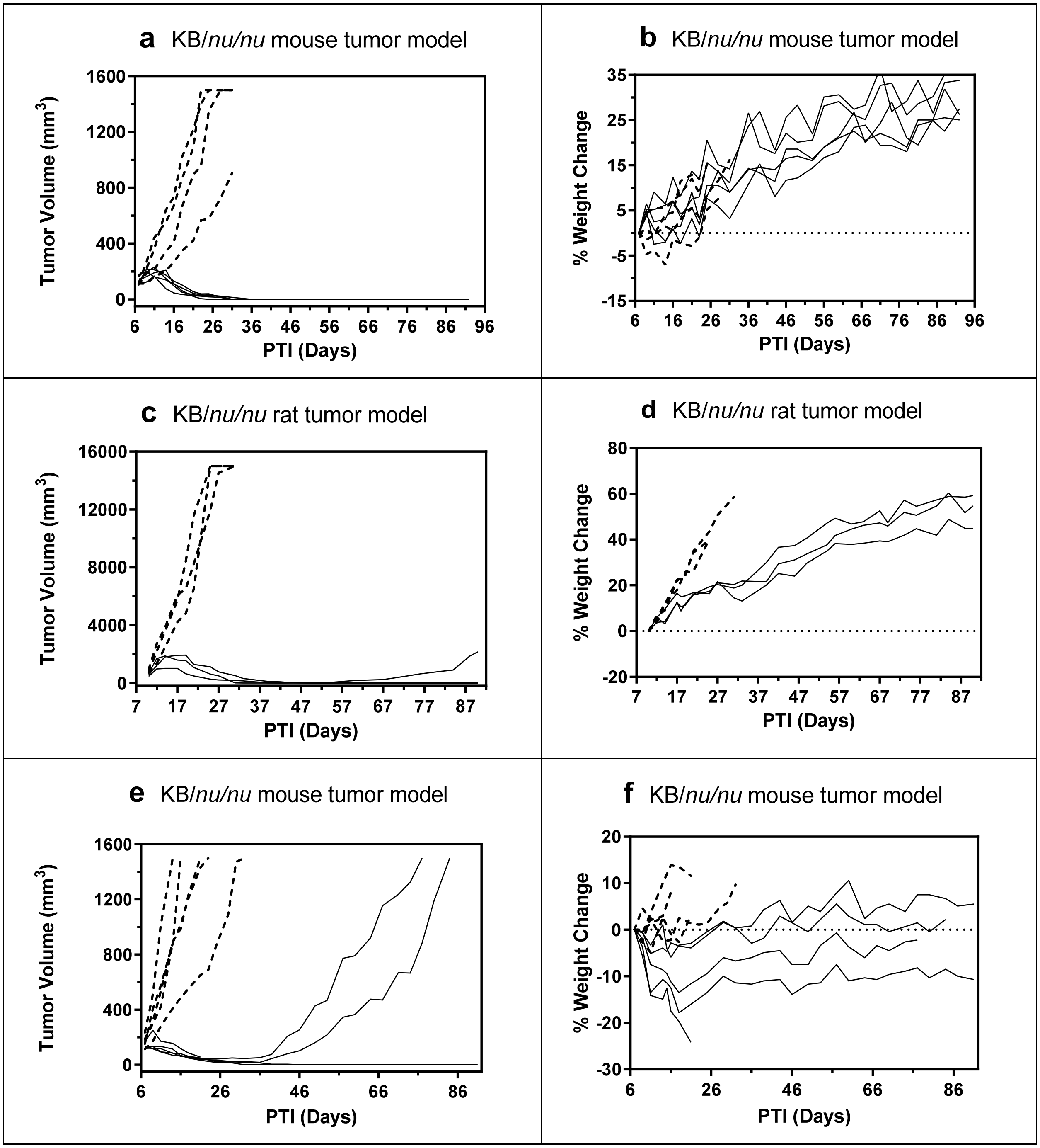

Supplementary Figure S1 Antitumor (a, c, e) and weight change (b, d, f) effects of EC2629 (a, b, c, d) and SJG-136 (e, f) on FR expressing KB tumor *nu/nu* mice (a, b, e, f) and rat (c, d) models. KB tumor cells were inoculated subcutaneously into *nu/nu* mice (1 x 10^6 cells) and rats (5 x 10^6 cells) and therapy started on randomized animals with tumors in the 111–168 mm^3 (mice; n = 5) and 411–704 mm^3 (rats; n = 3) range. (---), untreated controls; (—), EC2629, 0.3

μmol/kg (a, b) or 0.15 μmol/kg (c, d) or SJG-136, 0.3 mg/kg (e,f) in 0.05% Tween-80, SIW x 2 weeks. Each curve represents the growth of a single tumor in an individual mouse.

Supplementary Figure S2

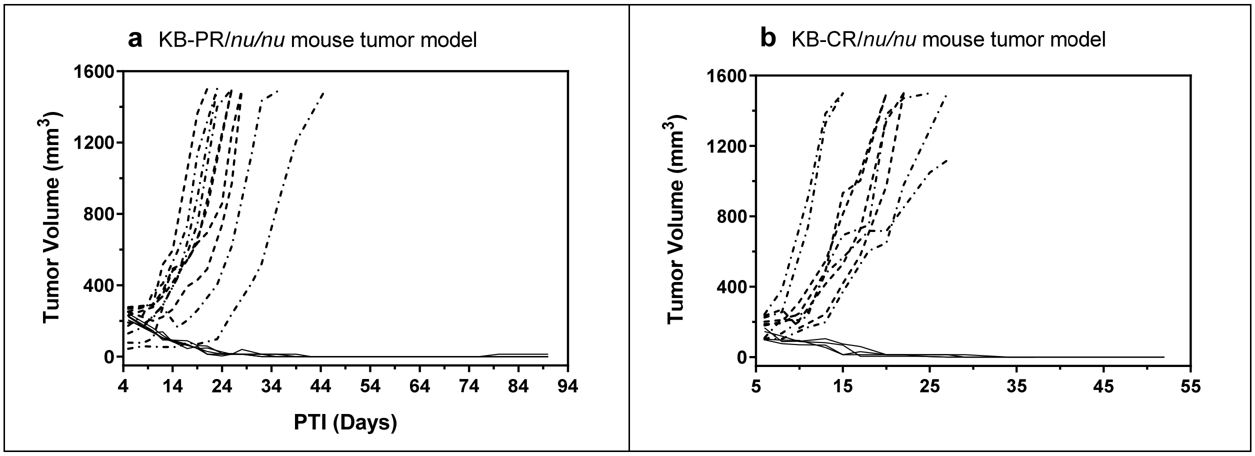

Supplementary Figure S2 Antitumor effects of EC2629 on FR expressing KB-PR (a) and KB-CR (b) tumors in *nu/nu* mice. KB-PR (a) or KB-CR (b) tumor cells (1 x 10^6) were inoculated subcutaneously into *nu/nu* mice and therapy started on randomized animals with tumors in the 205-243 mm^3 (a) and 98-168 mm^3 (b) range. (---), untreated controls; (-·-·-) (a), paclitaxel, 20 mg/kg, TIW x 2 weeks (b) cisplatin, 3 mg/kg, BIW x 2 weeks; (—), EC2629, 0.5 μmol/kg, SIW x 2 weeks. Each curve represents the growth of a single tumor in an individual mouse.

Supplementary Figure S3

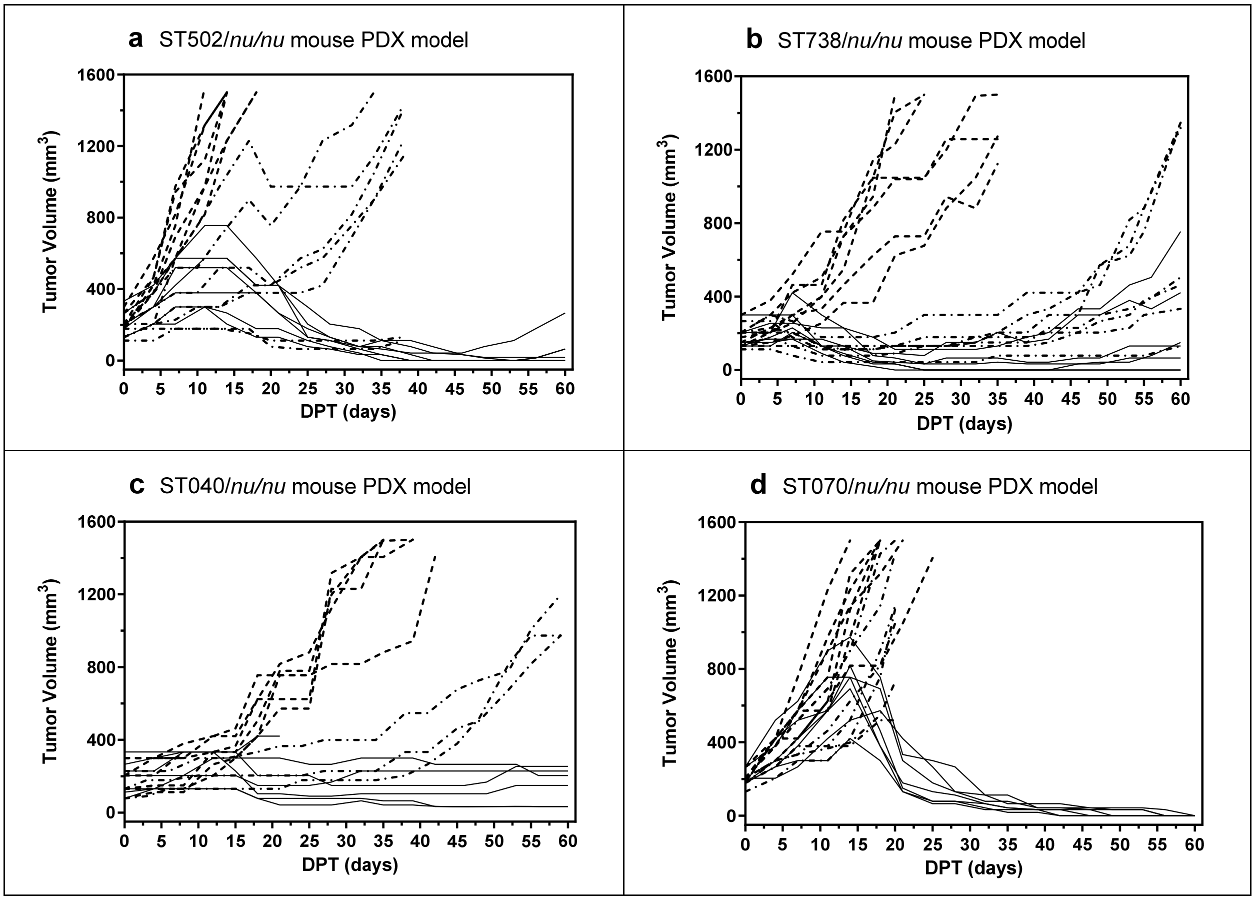

Supplementary Figure S3 Antitumor effects of EC2629 on patient derived triple negative breast ST502 (a), ST738 (b), endometrial ST040 (c) and ovarian ST070 (d) xenograft models in *nu/nu* mice. Tumor fragments harvested from host animals were inoculated subcutaneously into *nu/nu* mice and therapy started on randomized animals with tumors in the 112-333 mm^3 (a), 112 to 300 mm^3 (b), 78 to 333 mm^3 (c) and 131 to 266 mm^3 (d) range. (---), untreated controls; (-·-·-), eribulin mesylate, 1 mg/kg SIW x 2 weeks (a, b) or paclitaxel, 15 mg/kg, SIW x 2 weeks (c, d) (—), EC2629, 0.27 μmol/kg, BIW x 2 weeks (a,b,c,d). Each curve represents the growth of a single tumor in an individual mouse
